# Non-invasive assessment of pulsatile intracranial pressure with phase-contrast magnetic resonance imaging

**DOI:** 10.1371/journal.pone.0188896

**Published:** 2017-11-30

**Authors:** Geir Ringstad, Erika Kristina Lindstrøm, Svein Are Sirirud Vatnehol, Kent-André Mardal, Kyrre Eeg Emblem, Per Kristian Eide

**Affiliations:** 1 Department of Radiology and Nuclear Medicine, Oslo University Hospital—Rikshospitalet, Oslo, Norway; 2 Faculty of Medicine, University of Oslo, Oslo, Norway; 3 Department of Mathematics, Faculty of Mathematics and Natural Sciences, University of Oslo, Oslo, Norway; 4 The Intervention Centre, Oslo University Hospital, Oslo, Norway; 5 Department of Neurosurgery, Oslo University Hospital, Oslo, Norway; Universita degli Studi di Napoli Federico II, ITALY

## Abstract

Invasive monitoring of pulsatile intracranial pressure can accurately predict shunt response in patients with idiopathic normal pressure hydrocephalus, but may potentially cause complications such as bleeding and infection. We tested how a proposed surrogate parameter for pulsatile intracranial pressure, the phase-contrast magnetic resonance imaging derived pulse pressure gradient, compared with its invasive counterpart. In 22 patients with suspected idiopathic normal pressure hydrocephalus, preceding invasive intracranial pressure monitoring, and any surgical shunt procedure, we calculated the pulse pressure gradient from phase-contrast magnetic resonance imaging derived cerebrospinal fluid flow velocities obtained at the upper cervical spinal canal using a simplified Navier-Stokes equation. Repeated measurements of the pulse pressure gradient were also undertaken in four healthy controls. Of 17 shunted patients, 16 responded, indicating high proportion of “true” normal pressure hydrocephalus in the patient cohort. However, there was no correlation between the magnetic resonance imaging derived pulse pressure gradient and pulsatile intracranial pressure (R = -.18, P = .43). Pulse pressure gradients were also similar in patients and healthy controls (P = .26), and did not differ between individuals with pulsatile intracranial pressure above or below established thresholds for shunt treatment (P = .97). Assessment of pulse pressure gradient at level C2 was therefore not found feasible to replace invasive monitoring of pulsatile intracranial pressure in selection of patients with idiopathic normal pressure hydrocephalus for surgical shunting. Unlike invasive, overnight monitoring, the pulse pressure gradient from magnetic resonance imaging comprises short-term pressure fluctuations only. Moreover, complexity of cervical cerebrospinal fluid flow and -pulsatility at the upper cervical spinal canal may render the pulse pressure gradient a poor surrogate marker for intracranial pressure pulsations.

## Introduction

Idiopathic normal pressure hydrocephalus (iNPH) is a chronic condition characterized by gait ataxia, dementia and urinary incontinence[1] and can be treated successfully with shunt surgery. However, it remains a challenge to select which patients who will respond to such treatment. Unlike invasively obtained monitoring of *mean* intracranial pressure (ICP), over-night monitoring of *pulsatile* ICP has previously demonstrated to predict a beneficial shunt response in 9 of 10 patients [2, 3]. Increased pulsatile ICP is expected in iNPH while reduced pressure-volume capacity (reduced intracranial compliance) is a common feature [2, 4, 5].

The disadvantage with invasive monitoring of pulsatile ICP is the risk of severe complications such as cerebral bleeds and infections, which may occur in about 1%[6], and trends towards higher risk in iNPH patients who are older and with more comorbidity[7]. Given the useful role of invasive monitoring in patient care, there is an urgent need to develop alternative, non-invasive methods. There have been many attempts to apply phase-contrast MRI (PC-MRI) at intracranial locations to guide clinical decision making, particularly at the aqueduct level, but with varying degrees of success [8–13].

A non-invasively obtained marker for pulsatile ICP has been proposed, namely the PC-MRI-derived peak-to-peak pulse pressure gradient (MRI-dP), which is calculated from the cerebrospinal fluid (CSF) flow velocity change during one cardiac cycle at the upper cervical spinal canal[14]. Image acquisition is thus obtained in close proximity to the intracranial compartment; however, any pressure assessment at this level must be considered a surrogate for direct monitoring of pulsatile ICP.

Use of MRI-dP has previously shown promise in studies on computational fluid dynamics[15], baboons and humans[14, 16], as well as in patients with hydrocephalus[17, 18], Chiari malformation[19] and NPH[20]. MRI-dP has thus the potential to replace invasive monitoring in the pre-surgical diagnostic work-up of iNPH patients that are shunt candidates. Comparisons with invasive monitoring have though been few, and direct comparison with its invasive counterpart, pulsatile ICP, has so far been carried out in one baboon only[14].

It has previously been validated that over-night measurements of pulsatile ICP are comparable with measurements at daytime and are therefore neither sensitive to head position of the patient, unlike mean ICP [2].

The purpose of this study was therefore to assess the feasibility of MRI-dP by comparison with overnight invasive monitoring of pulsatile ICP in iNPH patients under clinical work-up for surgical shunting. We also compared MRI-dP derived from patients and healthy controls.

## Materials and methods

### Study population and design

The study and patient consent procedure was approved by the Institutional Review Board of Oslo University Hospital (07/5869) and Regional Ethics Committee (REC), South-East, Norway (S-07237). Each study participant was given written and oral information about the study and signed the consent form prior to inclusion in the study. All research was conducted according to the principles expressed in the Declaration of Helsinki. The institution that granted permission was Oslo University Hospital.

In this prospective study, a cohort of 34 consecutive patients with clinically probable iNPH, hospitalized within the department of neurosurgery as part of their preoperative diagnostic work-up, were enrolled. PC-MRI was done at the end of a routine MR image acquisition protocol and preceded invasive ICP monitoring performed during the same hospital admission. Additionally, four healthy controls were examined with PC-MRI acquisitions at different time points during one day.

### Clinical management

All patients were scored clinically using a clinical NPH grading scale [2], which assesses the combined severity of (I) gait disturbance, (II) urinary incontinence, and (III) dementia. Each of the three variables is graded from 1 to 5, giving a possible worst score of 3 and best score of 15. The decision for shunt surgery was based on a combination of clinical assessment, radiological assessment and results of continuous ICP monitoring [2].

The shunt response was defined as an increase by at least 2 points on the NPH grading scale, and clinical score was assessed at regular intervals, 3, and 6–12 months following shunting.

### ICP monitoring

**Fig 1:**
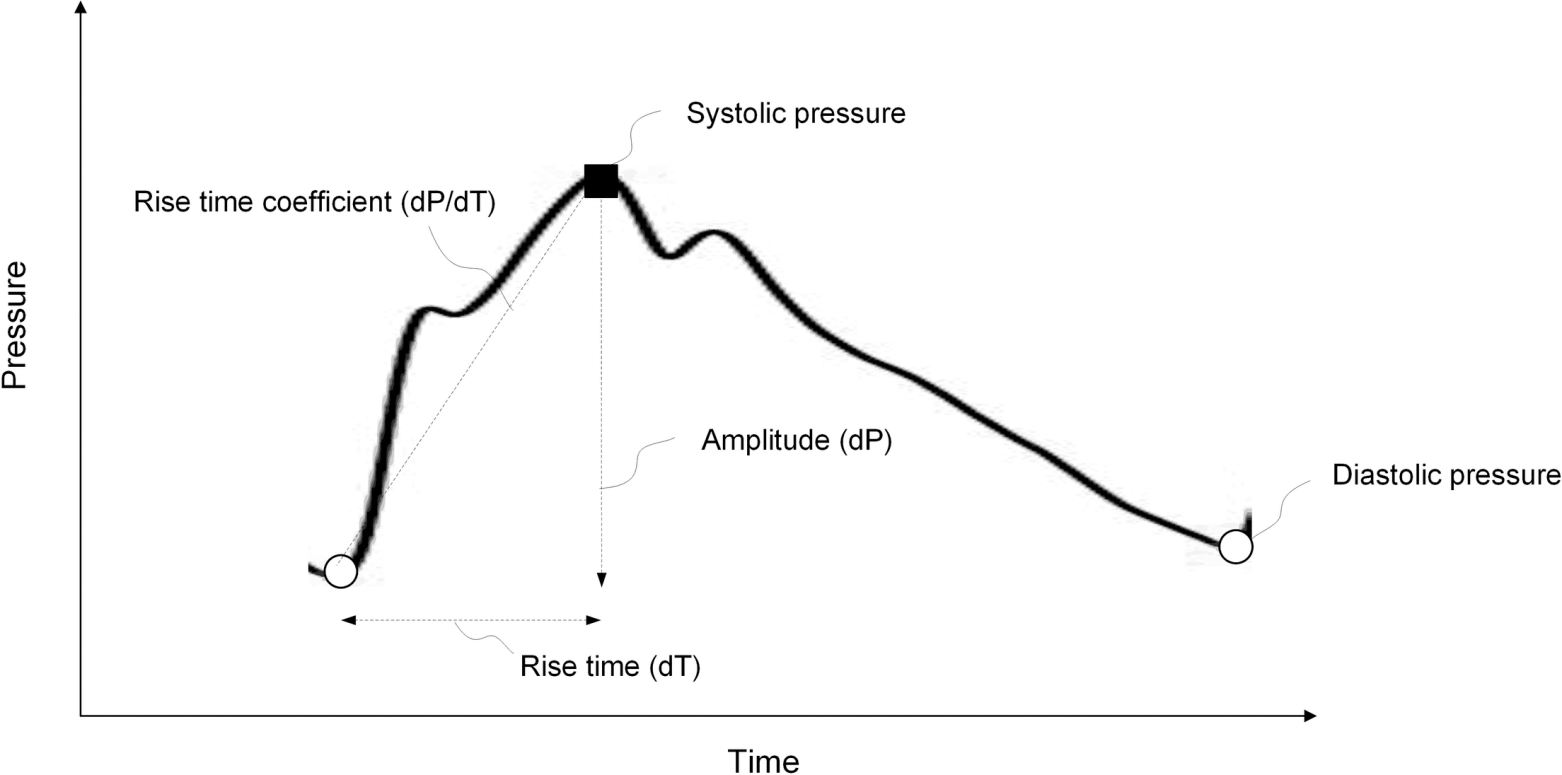
Single cardiac-induced ICP wave. One single ICP wave is illustrated with its individual parameters. We measured pressure in mm Hg; with a heart rate of 60, the duration of a single wave is about 1 second. The mean ICP wave amplitude (MWA) is determined as the average of amplitudes from single ICP waves during consecutive 6-second time intervals.

The procedure for continuous overnight ICP monitoring has previously been described[2]. In short, an ICP sensor is placed in the frontal lobe of the brain through a small burr hole in the skull, using local anesthesia. The monitoring is carried out in the patient ward with the patient in the supine position (equal to patient position at PC-MRI) using a computerized system (Sensometrics AS, dPCom, Oslo) for automatic identification of individual cardiac-induced single ICP waves. The amplitude of the ICP wave (pulsatile ICP) is defined as the pressure difference between the systolic maximum and diastolic minimum pressures (Fig 1). Pulsatile ICP expressed by the mean ICP wave amplitude (MWA) is determined during consecutive 6-second time intervals, and over the entire observation period. Mean (static) ICP is the average of absolute ICP relative to a zero pressure level. The MWA was used for selection to shunting according to the department routine [2]. MWA in average ≥4 mmHg and/or percentage of MWA ≥5 mmHg in ≥10% of recording time was set as threshold levels for shunting.

### PC-MRI acquisition protocol

For the iNPH patients, all PC-MRI images were obtained in a Philips 3 Tesla Achieva system (Philips Medical Systems, Best, The Netherlands) with a 16-channel head coil and an acquisition plane perpendicular to the upper cervical spinal canal at level C2, which is typically the closest level to the intracranial compartment where the assumption of the spinal canal as a rigid, cylindrical tube can be made when estimating MRI-dP. The PC-MRI scanning parameters were as follows: Repetition time (TR) and echo time (TE) was shortest possible, typically TR/TE = 16/11 ms, pixel size 0.56 x 0.56 mm^2^ to 0.63 x 0.63 mm^2^, slice thickness 7 mm, velocity encoding 6 cm/sec, and 32–40 phases with retrospective peripheral cardiac gating based on pulse oximeter plethysmography. Potential sources of inaccuracy in velocity measurements, such as eddy currents, Maxwell terms and gradient field non-linearities[21] had been corrected for as far as possible by the vendor of the scanner before the study, and also sought minimized during the study by optimal patient positioning. Duration of the PC-MRI acquisition was approximately 6 minutes. MR imaging always preceded any surgical shunting procedure.

The healthy controls were scanned in a Philips 3 Tesla Ingenia system (Philips Medical Systems, Best, The Netherlands) with a 32 Channel head coil, and with identical acquisition plane,—level and—parameters as for the patients. Each healthy control was scanned with four PC-MRI acquisitions over at least two different time points during one day and with duration between time points of approximately two hours. Acquisitions within one time point were obtained consecutively without pause. All patients and controls were studied at daytime between 8 a.m. and 4 p.m.

**Fig 2:**
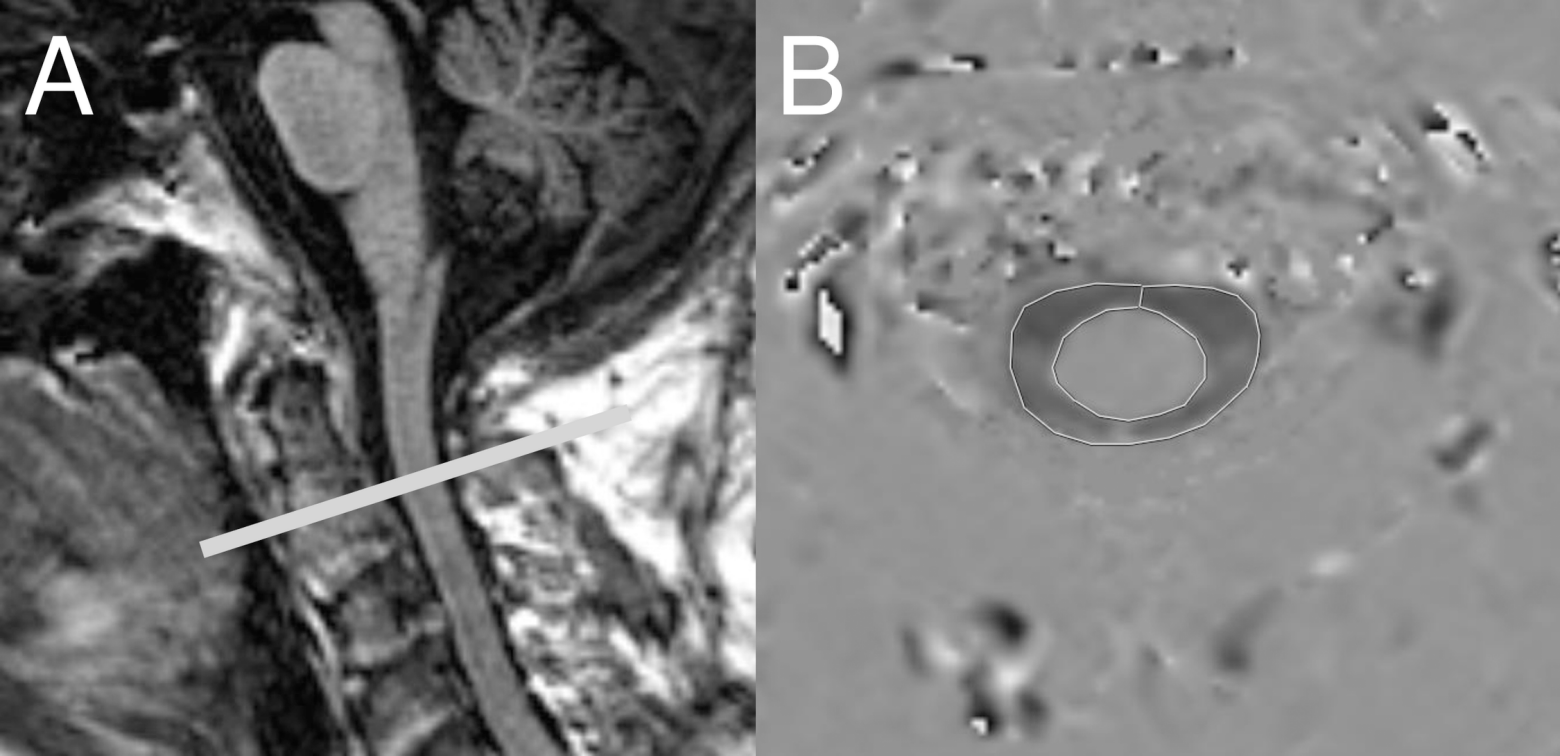
**(A) Level of PC-MRI acquisitions.** PC-MRI was performed perpendicular to the cervical spinal canal at level C2, which is typically the closest level to the intracranial compartment where the assumption of the spinal canal as a rigid, cylindrical tube can be made when estimating MRI-dP. **(B) ROI outlining.** The subarachnoid space was manually defined from phase images with a region of interest (ROI).

Fig 2 A demonstrates the slice orientation and level for all PC-MRI acquisitions.

### PC-MR image outlining

The subarachnoid fluid compartment surrounding the spinal cord was manually defined from phase images with a region of interest (ROI) by use of dedicated software, nordicICE^®^ (Fig 2B) (NordicNeuroLab AS, Bergen, Norway) and was performed by an experienced neuroradiologist, blinded to NPH score and ICP data.

### Pixel transformation and treatment of aliasing

Recorded velocities were converted by linear transformation from pixel values to centimeters per second by applying the velocity encoding using MATLAB^®^ (Mathworks, Natick, United States). Aliased velocities were treated with a filter that was activated if the temporal variation between two consecutive time steps of the examined pixel velocity exceeded a threshold value of 1.1 times the VENC. The aliased pixel was replaced by *v* = *v*_a_ ± 2 × *V*_*enc*_, where *v* is the filtered velocity, *v*_a_ is the aliased pixel value and *V*_*enc*_ is the velocity encoding.

### Computation of pressure gradients

The method for computation of MRI-dP (mm Hg/cm) has been described in detail previously[14].

Mean velocities were computed by summarizing velocities at each pixel over the domain, and dividing the sum with the number of pixels.

MRI-dP was computed according to Navier-Stokes equations for incompressible and Newtonian fluid. Velocities were recorded across an axial (x,y) plane, which reduced the Navier-Stokes equations to the z-component. Further, the flow was assumed parallel to rigid walls of a cylindrically shaped tube and the convective term was neglected. The resulting equation for the pressure gradient is given by

$$\frac{\partial p}{\partial z}=-\rho \frac{\partial V}{\partial t}+\mu \left(\frac{\partial^{2}V}{\partial x^{2}}+\frac{\partial^{2}V}{\partial y^{2}}\right)$$

where the first term on the right-hand side describes the transient inertial forces and the second term describes the viscous forces in the flow. *ρ* = 1.0007 g/cm^3^ is the density of the CSF, and μ = 1.1 cP is the dynamic viscosity, and which are considered not to be affected by the iNPH condition.

**Fig 3:**
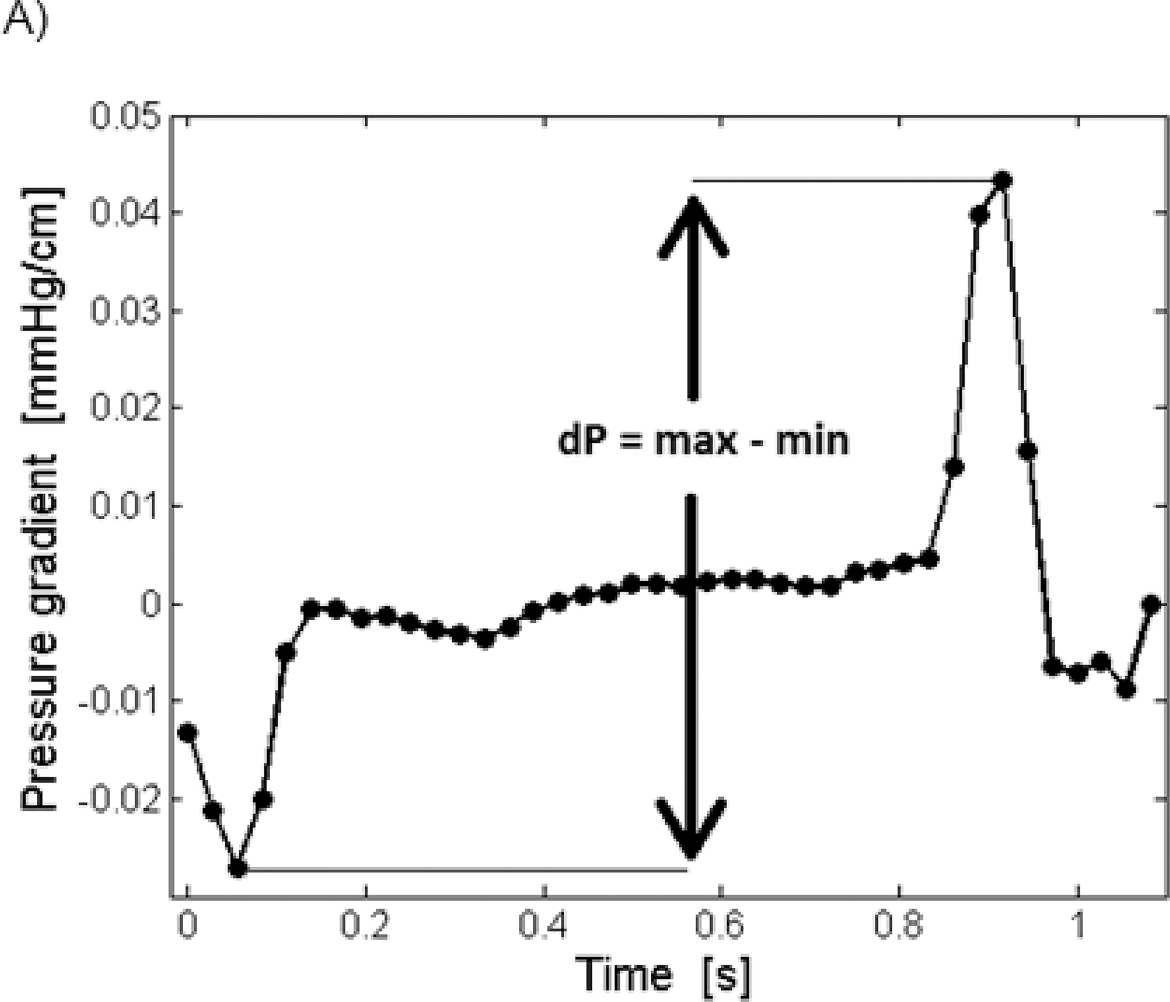
The PC-MRI derived pulse pressure gradient (MRI-dP). MRI-dP is calculated as the difference between the maximum and minimum pressure gradient within the cycle.

Pressure gradients for all pixels in the ROI were computed before they were averaged over the domain, resulting in one mean pressure gradient for each period. MRI-dP was calculated as the difference between the maximum and minimum pressure gradient within the cycle (Fig 3).

### Statistical analysis

ICP data, counting several thousands of observations from over-night monitoring, were assumed normally distributed, and presented in each patient with mean and standard deviation. For patients and healthy controls (n = 22 and 4, respectively), PC-MRI data were assumed not normally distributed and presented with median and range. Correlations were determined by Pearson correlation coefficient, and agreement between methods was evaluated with a Bland-Altman plot. Comparisons between patients and healthy controls were performed with Mann-Whitney U test. One-way ANOVA was used to compare means of more than two groups. Reliability (absolute agreement, two-way mixed) of multiple quantitative measurements within individuals was estimated with the intraclass correlation coefficient (ICC). The significance level was set to 0.05. Statistical analysis was performed using SPSS Statistics version 20 (IBM Corporation, Armonk, NY, US).

## Results

### Study population

Of 34 enrolled patients, 12 were excluded from the study due to motion artifacts at PC-MRI (5/12), termination of the examination by the patient or MRI technician before completion (5/12), PC-MRI obtained at a suboptimal level (1/12) or image artifacts (1/12). Thus, 22 patients were included. Shunt surgery was performed in 17 of these, of which 16 (94%) were clinical responders. Other patient data are given in Table 1 and S1 File. Age and gender of four healthy controls are also presented in Table 1.

**Table 1:** Demographic information about patients and healthy controls.

***NPH patients***	
Number	22
Age (yrs)	71 (45–84)
Gender (F/M)	8/14
BMI (kg/m^2^)	25.4 (20.3–30.9)
Cardiovascular co-morbidity (n)	12
Treatment	
Shunt (Responders/Non-responders)	17 (16/1)
No shunt	5
***Healthy individuals***	
Number	4
Age (yrs)	31 (23–40)
Gender (F/M)	1/3

Results presented as numbers for categorical data and as median (range) for continuous data.

### ICP scores

**Fig 4:**
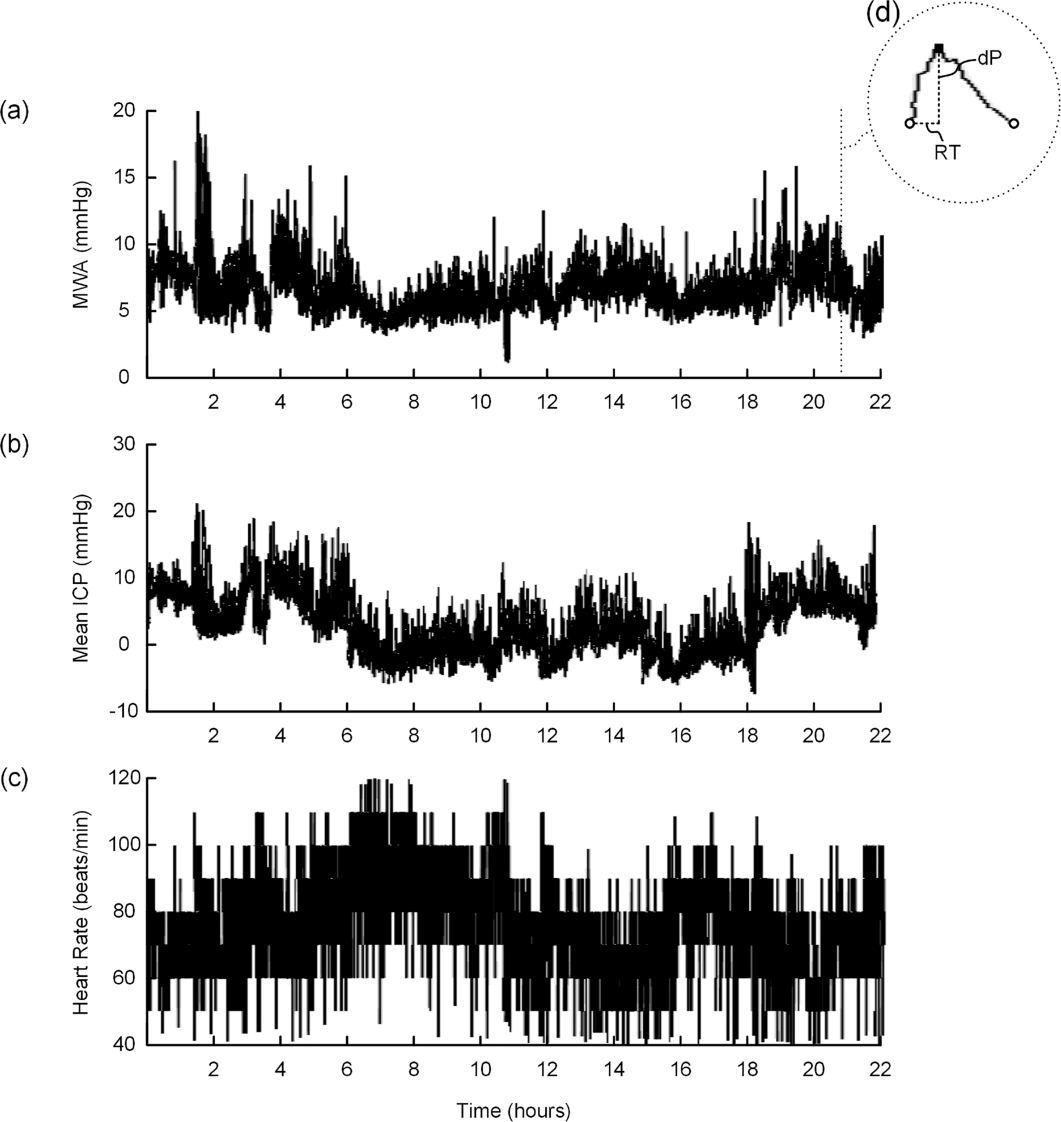
Trend plots of (a) MWA (6.6±1.8 mmHg), (b) mean ICP (2.8±4.8 mmHg), and (c) HR (76.2±13.7 beats/min) for patient 20, who underwent prolonged invasive monitoring, illustrating the variation in the ICP-derived parameters over time. In (d) is shown one individual cardiac-beat induced ICP wave with its amplitude (dP) and rise time (RT).

A summary of the ICP data from the iNPH patients are given in Table 2, and the full dataset from the patient cohort is presented in S1 Table. The coefficient of variation (CV = standard deviation/mean) illustrates the span of fluctuations in measured pulsatile and static ICP during overnight monitoring. Overnight monitoring of pulsatile and static (mean) ICP demonstrated large fluctuations, where CV was 26% (12, 41) and 128% (19, 5600), respectively (median and range). Fig 4 exemplifies recordings from a study patient demonstrating pulsatile ICP expressed by MWA (a), static ICP expressed by mean ICP (b) and heart rate (c) as a function of time. For patient specific measurements, MWA was to a very limited degree affected by HR, as the association between pulsatile ICP (MWA) and heart rate (HR) was low with median R = .02 (S1 Table).

**Table 2:** Summary of ICP data from iNPH patients.

Patients	Observations (6-s windows)	MWA (mm Hg) (mean)	MWA (mm Hg) (SD)	CV_MWA_ (%)	Mean ICP (mm Hg) (mean)	Mean ICP (mm Hg) (SD)	CV_MeanICP_ (%)
All (n = 22) (median with range)	13.283 (1501, 21397)	4.4 (2.2, 7.3)	±1.1 (±.5, ±2.2)	26 (12, 41)	2.8 (-4.5, 14.5)	±4.4 (±1.9, ±11.8)	128 (19, 5600)

**ICP:** intracranial pressure

**iNPH:** idiopathic normal pressure hydrocephalus

**MWA:** mean ICP wave amplitude (pulsatile ICP)

**CV:** coefficient of variance (SD/mean)

**SD:** standard deviation

### PC-MRI data from patients and healthy controls

A summary of the PC-MRI data from patients and healthy controls is shown in Table 3, and an extended set of data from the PC-MRI studies of iNPH patients and healthy controls are presented in S2 and S3 Tables, respectively, and in S2 File.

**Table 3:** PC-MRI data from patients and healthy controls (median with range).

	Patients (n = 22)	Healthy (n = 4)	P-value*
MRI-dP (mmHg/cm)	.041 (.015, .083)	.053 (.031, .093)	.39
ROI area (cm^2^)	1.74 (1.20, 3.50)	1.26 (.77, 1.58)	.016
Number of pixels	446 (306, 895)	323 (198, 405)	.016
Heart rate (/min)	68 (52, 92)	61 (49, 69)	.25

**PC-MRI:** phase-contrast magnetic resonance imaging

**MRI-dP:** MRI-derived peak to peak pulse pressure gradient

**ROI:** region of interest

*Mann-Whitney U-test.

MRI-dP at level C2 was not different in iNPH patients and healthy subjects (P = .39). Only area of the subarachnoid space (ROI area and hence number of pixels) differed between groups (P = .016).

### Comparison of patient ICP scores and MRI-dP

**Fig 5:**
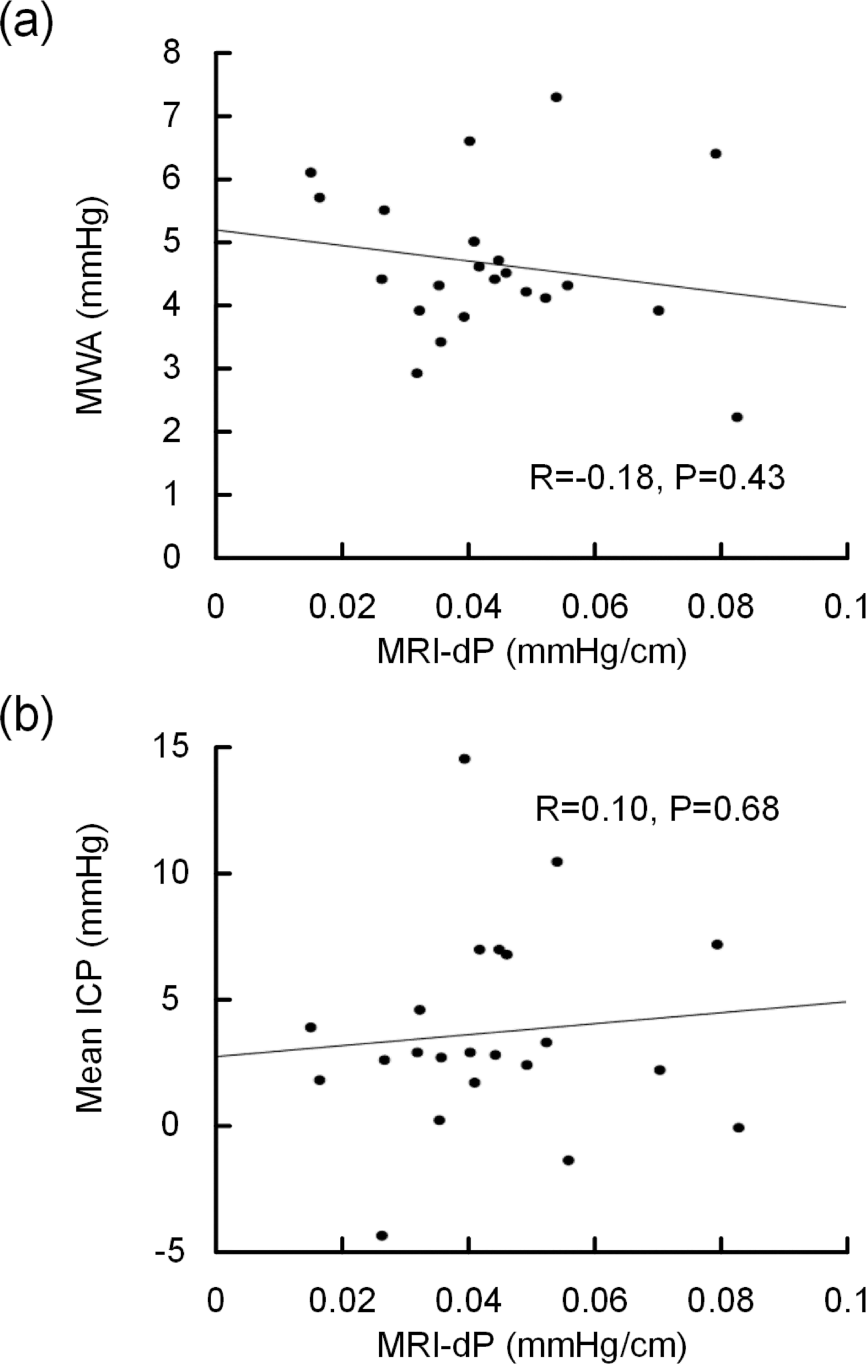
MRI-dP and ICP parameters. The association between MRI-dP and standardized over-night, invasive monitoring (11 p.m. to 7 a.m.) of (a) pulsatile ICP (MWA) and (b) static ICP (mean ICP).The Pearson correlation coefficient (R) and significance level are presented for each plot.

**Fig 6:**
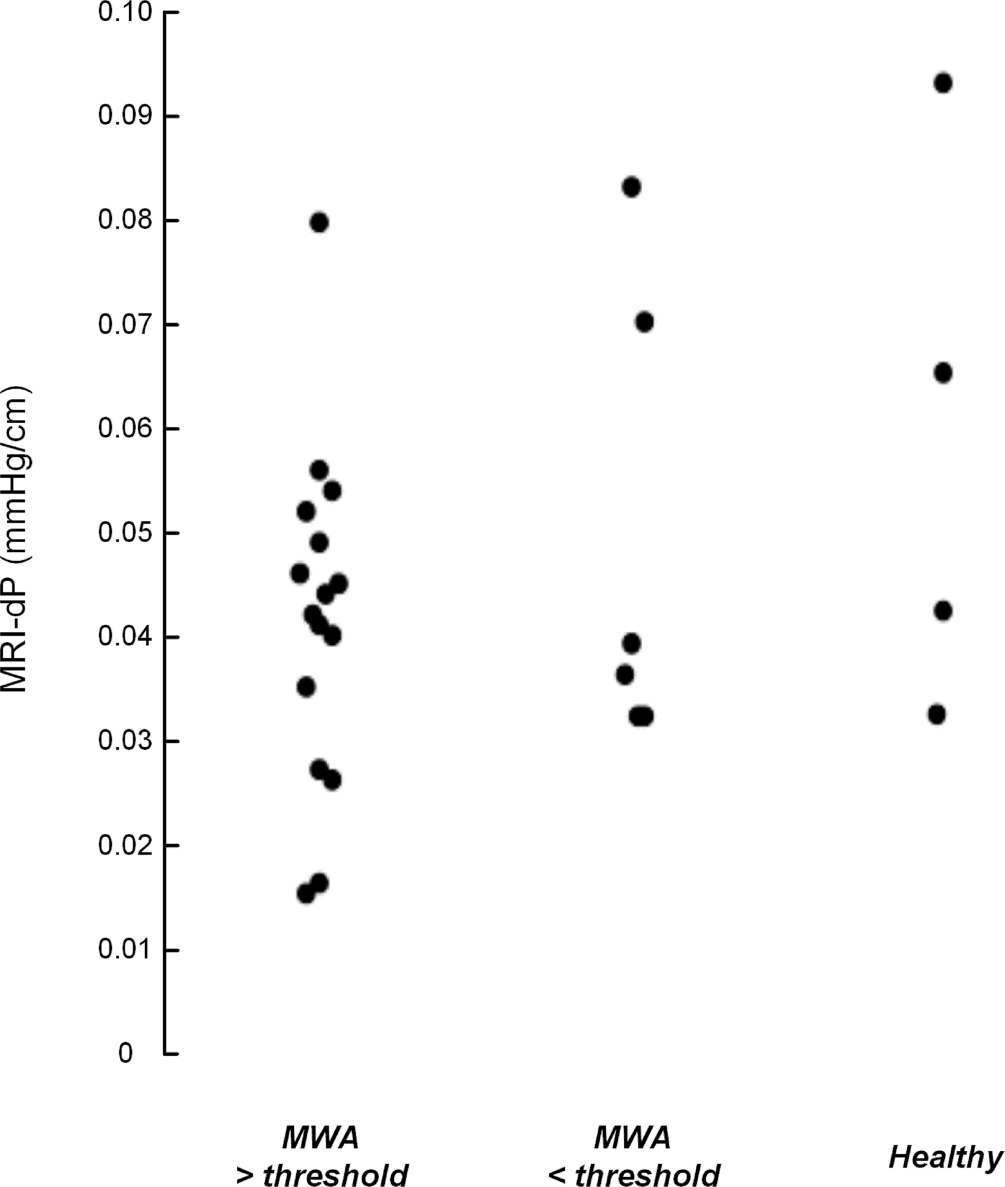
MRI-dP and pulsatile ICP (MWA) thresholds for shunting in iNPH. The MRI-dP values are shown for iNPH patients with preoperative MWA values either above (n = 16) or below (n = 6) the thresholds used for selection of patients for shunting, and also for the healthy reference subjects. There were no significant differences between groups (P>0.44).

There were no associations between invasively measured pulsatile ICP and the non-invasive assessment of MRI-dP (R = -.18, P = .43) (Fig 5A) or mean ICP and MRI-dP (R = .10, P = .68) (Fig 5B). Moreover, the MRI-dP did not differ between individuals with MWA above or below established thresholds for shunting (P = .97), or healthy controls (P = .44) (Fig 6).

### Heart rate during PC-MRI and ICP monitoring

**Fig 7:**
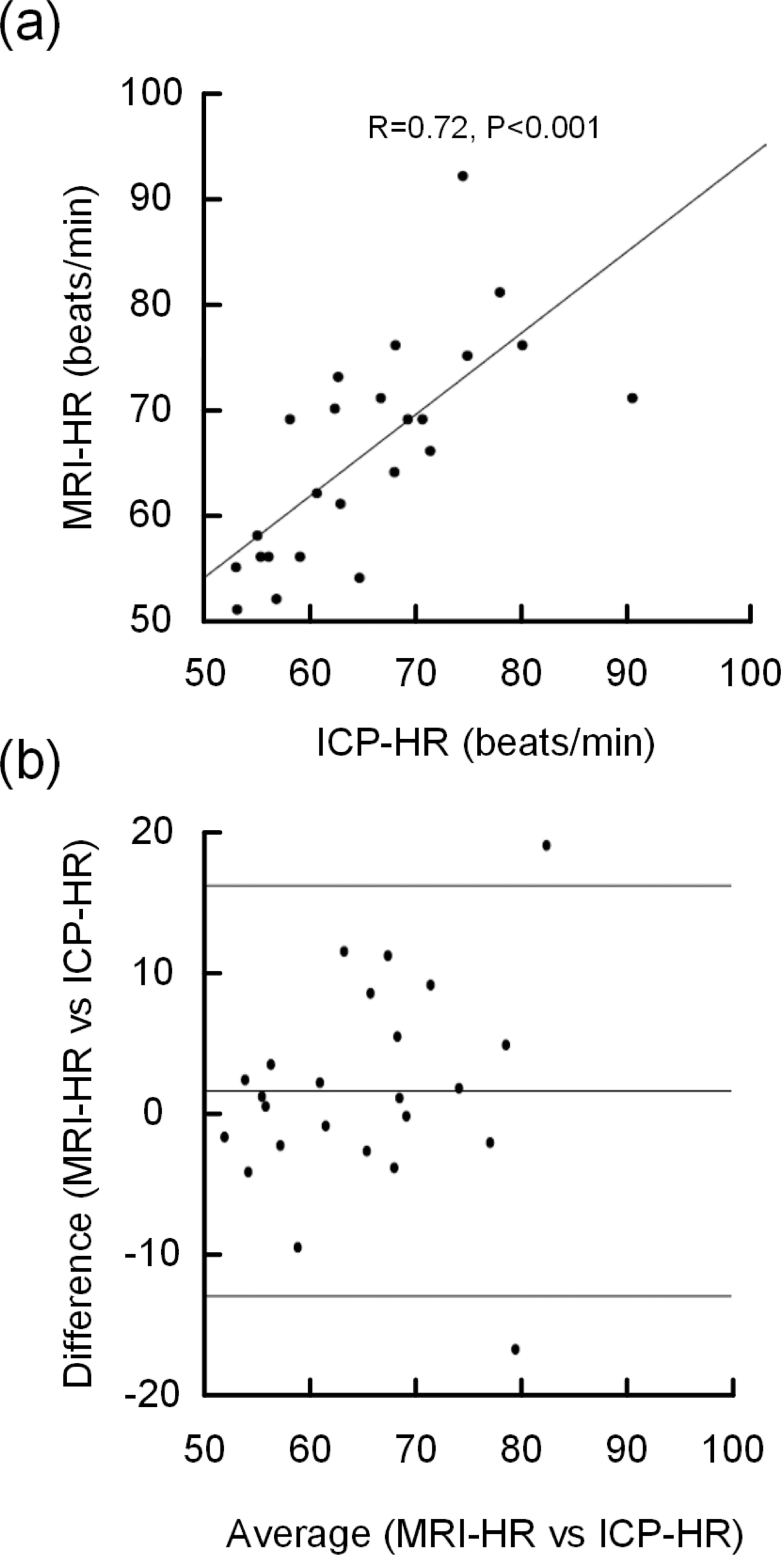
HR from PC-MRI and ICP monitoring. (a) The association between MRI-derived HR and ICP-derived HR, Pearson correlation coefficient (R) and significance level. (b) Bland-Altman plot of all MRI- and ICP-derived HR observations. The line in the middle is the mean difference (2.3 beats/min) and the upper and lower lines represent mean+2SD (standard deviations; 15.0 beats/min) and mean– 2SD (-10.4 beats/min), respectively.

There was a high correlation between heart rates (HR) from PC-MRI and invasive ICP monitoring (R = .71, P = .001) (Fig 7A), and the Bland-Altman plot revealed no systematic differences in HR registered during invasive ICP monitoring and PC-MRI (Fig 7B).

## Discussion

Invasive monitoring of pulsatile ICP has previously proven to be a precise tool to select which iNPH patients who will respond to surgical shunting. In this study, we tested the utility of PC-MRI to non-invasively assess a surrogate marker for pulsatile ICP, MRI-dP, obtained at level C2. MRI-dP did not correlate with invasively obtained pulsatile ICP from over-night monitoring, and did not discriminate iNPH patients from healthy subjects.

Increased pulsatile ICP due to reduced intracranial compliance is common in iNPH [7], and patients with this disease should therefore be well suited for a study of non-invasive assessment of pulsatile ICP using PC-MRI. In the present study cohort, increased pulsatile ICP expressed by MWA above threshold level was diagnosed in 17 of 22 patients. Surgical shunting yielded a clinical improvement in 16 of 17 treated patients, indicating a high proportion of what may be considered “true iNPH”.

MRI-dP has previously been applied in a proposed method for non-invasive estimates of static (mean) ICP [14]. With this method, the elastance index is estimated by dividing MRI-dP by the per cardiac cycle total intracranial volumetric change (MRI-dV), i.e. MRI-dP/MRI-dV. The linear relationship between elastance and ICP is expected owing to the monoexponential relationship between intracranial volume and pressure[22]. Precise assessment of MRI-dP is thus an important precondition for such estimation of static ICP as well.

Should a non-invasive measure such as MRI-dP be valid, it would have to reflect its gold standard of invasive measurement (pulsatile ICP). Both pulsatile and static (mean) ICP demonstrated in this study large fluctuations during the overnight monitoring, and this phenomenon is also a determining factor as to why clinical decisions based on monitoring of pulsatile ICP are made after analysis of observations from an extended time span (Fig 4). A PC-MRI based method applied to obtain measures of pulsatile ICP through a surrogate parameter like MRI-dP from a short time interval should therefore be interpreted with great care. One previous study could not demonstrate different MRI-dP between Chiari malformation type 1 patients and controls[23], even though elevated pulsatile ICP is frequently observed when measured over-night[24].The aqueductal stroke volume is another, MRI derived, CSF velocity based parameter obtained from a short time interval, which has been proposed to serve as a surrogate for ICP recordings[8], and were also unable to demonstrate any association with its invasive counterpart[13, 25].

There may be several reasons as to why MRI-dP derived from one simple PC-MRI acquisition does not reflect pulsatile ICP measured overnight. First, MRI-dP is based on acquisition of CSF velocity change at the upper cervical spinal canal, and is thus a surrogate marker for intracranial pressure change. A simplified model of a rigid, cylindrically shaped tube is desirable in order to make valid assumptions in fluid dynamic equations used in post-processing of PC-MRI data, where attempts made intracranially, with much more complex anatomy, are mostly restricted to the Sylvian aqueduct. However, even though the upper spinal canal is anatomically in close proximity with the intracranial compartment, the geometry is fundamentally different, and from computational fluid flow dynamics, it has been demonstrated that lower pressure values can be expected at level C2 than in the posterior fossa due to pressure gradients that become steep in the cervical canal[26].

Secondly, the CSF flow at level C2 is the result of a dyssynchronous pressure pulsation causing a pulsating cranio-cervical pressure gradient pumping CSF in and out of the cranial vault. The pressure gradient estimated from MRI is relatively small, and in this study, less than 0.1 mm Hg per cm in all patients. This magnitude corresponds well with previous studies. From numerical studies, where the CSF flow has been modeled in rigid and impermeable surroundings, the fluid flow has been predominated by velocities orthogonal to the axial plane, and the observed pressure gradients at level C2 in healthy subjects is also less than 0.1 mm Hg per cm[27, 28]. Computations that take into account the elasticity of the cervical spinal cord, tonsillar motion and the presence of nerve roots and denticulate ligaments, all predict pressure gradients of similar magnitudes[29–31]. Hence, the pressure gradient between the cervical and cranial compartments is but a fraction of the total pressure pulsation.

**Fig 8:**
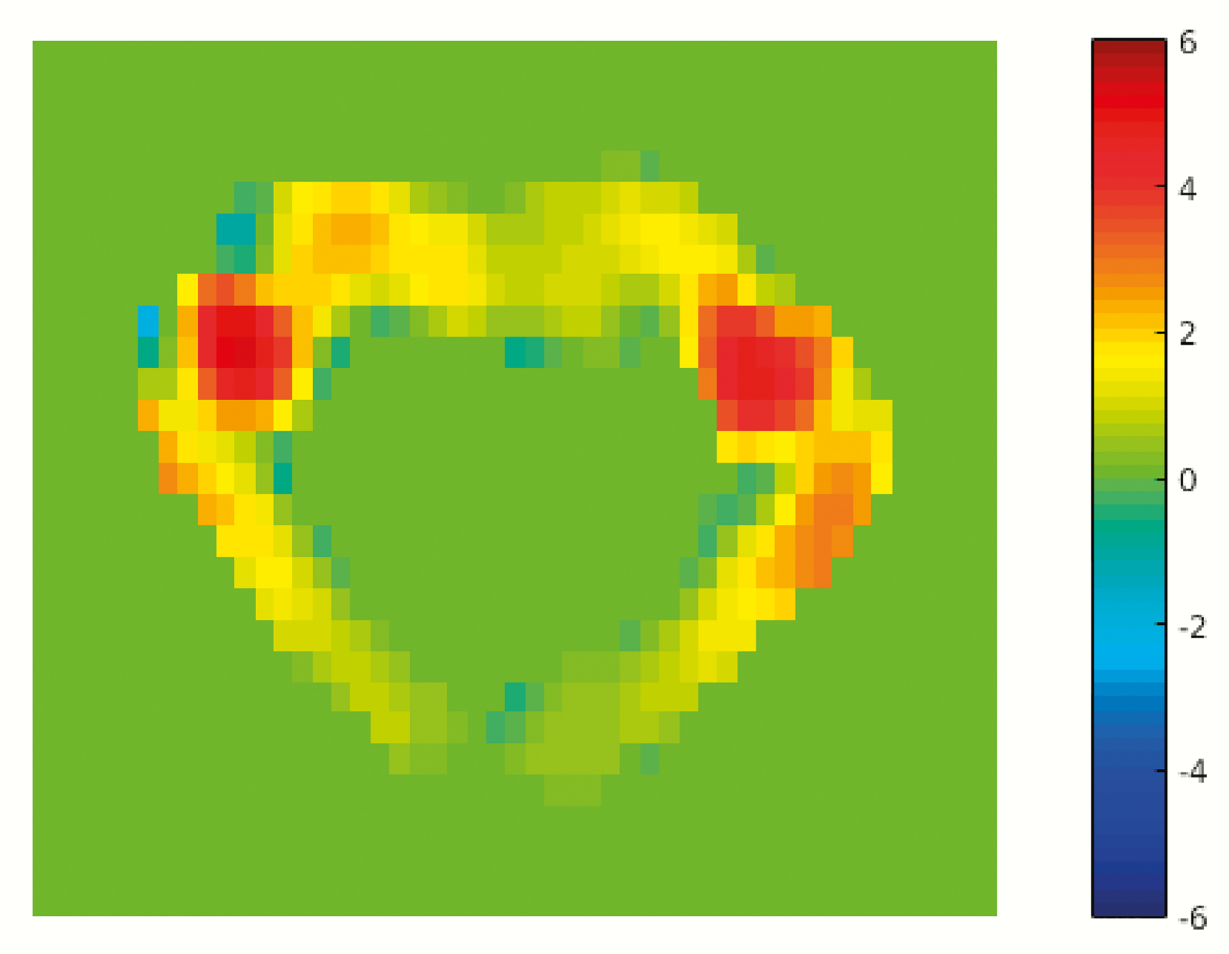
Heterogeneity of CSF flow velocities. Patient 4, demonstrating spatial variations in flow velocity within the ROI at one time step (t = 0.70 s). The color bar represents velocities in cm/s.

Moreover, the pressure gradient computation in this study is based on a number of simplifying assumptions such as rigid and impermeable surroundings and laminar flow. CSF flow patterns are, however, complex due to the anatomy of the cervical subarachnoid space[26], and flow velocities in axial sections of the spine have been shown to have non-uniform distributions throughout the cardiac cycle as demonstrated from computational simulations[28]. In our study, the pixel-by-pixel analysis revealed a wide dispersion of flow velocities from within the same PC-MRI slab (Fig 8). Averaging of flow velocities from a ROI defining the CSF space therefore does not fully describe the diversity or complexity of velocity changes that occurs through a section of the cervical spine during one cardiac cycle.

Interestingly, it may be noted that the healthy control with smallest CSF ROI area also had the largest MRI-dP among controls (No. 2 in S3 Table), and larger than in many iNPH patients with invasively proven increased pulsatile ICP (S2 Table). Previously, the inverse and almost linear relationship between MRI-dP and CSF flow area has been reported[14], and very steep pressure gradients have also been demonstrated in Chiari patients with tonsillar crowding at the foramen magnum[32]. For the patient group in our study, there was a trend towards an inverse correlation between MRI-dP and area of the subarachnoid fluid area at level C2 (ROI area), but this did not reach significant levels (R = -.32, P = .15). However, we notice that measuring reliability (ICC) was almost identical for MRI-dP and ROI area measurements in healthy controls. Thus, while MRI-dP should ideally be a marker of pulsatile ICP only, it may be hypothesized that a narrow CSF space surrounding the spinal cord may influence directly on MRI-dP. However, CSF area is already implicitly accounted for when estimating pressure based on the Navier-Stokes equation.

Finally, but not least, recent studies have demonstrated respiration to have a major influence on CSF flow [33, 34]. With cardiac gated PC-MRI, which can be considered to represent current state-of-the-art methodology in PC-MRI based measurements of intracranial and intraspinal CSF flow quantification, only cardiac beat induced CSF flow is detected. Patient breathing is not controlled for.

The invasive ICP measurements of this study were not performed synchronously with PC-MRI, but overnight, as standardized according to previous empirical observations supporting this routine. In principle, our data do therefore not in principle contradict that MRI-dP may reflect pulsatile ICP in real time. The comparison with invasive monitoring for a prolonged time period is, however, highly relevant, because clinical decision making is rarely based on ICP monitoring from a time interval as short as the duration of a PC-MRI acquisition. It has previously also been validated that over-night and daytime measurements of pulsatile ICP are comparable, unlike measurements of static ICP[2].

Another limitation was that technically adequate PC-MRI was obtained in less than 2/3 of enrolled patients, while all examinations in healthy subjects were successful. NPH patients are typically elderly and suffer from discomfort during long MRI scan times, and the experimental PC-MRI acquisitions were performed towards the end of the imaging protocol. In iNPH, dementia is a common feature, which may be a challenge to patient cooperation throughout the scan. MRI-dP from PC-MRI may therefore seem more feasible in subjects that cooperate well rather than in patients where compliance to instructions from the MR technician can be a challenge. The inability to exam patients with the poorest level of cooperation skills may have introduced bias to the study, because more severely affected patients might have been more likely to be excluded.

Moreover, the velocity encoding gradient (venc) was set at a low level for the PC-MRI acquisitions in patients and healthy controls. Because of flow heterogeneities, flow aliasing was quite frequent. We consider, however, this potential source of error to be small because of the aliasing correction procedure and that flow velocity values from each time point represent the average of velocities from the entire region of interest. Pixels with any inaccuracies of flow velocity measurements should therefore not have contributed substantially to the calculated MRI-dP.

As for PC-MRI based measurements of CSF flow in general, a major concern with the technique would be the extraction of data from a limited time interval to make a diagnosis in patients where well-known physiological fluctuations are present. In the present study, all PC-MRI exams may be assumed to have been performed within normal physiological boundaries, as they were obtained within a normal range for heart rate (Tables 2 and 3), and the statistical analysis did not reveal any systematic differences in heart rate (HR) between PC-MRI and ICP monitoring. This indicates that influence from patient stress and discomfort was not very different during PC-MRI and ICP monitoring, and this source of bias in comparing the methods should therefore have been modest.

## Conclusions

Invasively obtained pulsatile ICP with level above thresholds has previously been demonstrated to predict a beneficial shunt response in 9 of 10 patients with iNPH. In our study cohort of iNPH patients, patient selection with this method yielded a beneficial shunt response in 94%. However, pulsatile ICP was not associated with its non-invasive counterpart, the PC-MRI derived MRI-dP. Moreover, MRI-dP did not discriminate between patients with either preoperative values of pulsatile ICP above or below thresholds, and neither patients from healthy subjects. MRI-dP obtained at level C2 was therefore not found feasible to replace invasive monitoring of pulsatile ICP in selection of iNPH patients for surgical shunting. Unlike invasive, overnight monitoring, MRI-dP comprises short-term pressure fluctuations only. Moreover, complexity of cervical CSF flow and -pulsatility at the upper cervical spinal canal may render MRI-dP a poor surrogate marker for intracranial pressure change.

## Supporting information

S1 Table ICP-data: Patients. Results from ICP monitoring in each of the iNPH study patients.
(DOCX)

S2 Table PC-MRI data: Patients. Results from PC-MRI in each of the iNPH study patients.
(DOCX)

S3 Table PC-MRI data: Healthy controls. Results from PC-MRI in each healthy subject at each time point. PC-MRI exams (MRI-1 and MRI-2) within one time point (e.g. “Time1”) were performed with the possible closest proximity in time. The distance in time between two time points (e.g. “Time1” and “Time2”) was approximately two hours.
(DOCX)

S1 File Patient data. Extended set of clinical patient data.
(XLS)

S2 File Pressure gradients and mean velocities. Calculated pressure gradients and mean velocities from single time points are given for all patients and healthy controls.
(ZIP)
