# Supplementary material for: Non-invasive assessment of pulsatile intracranial pressure with phase-contrast magnetic resonance imaging
Source: PLoS One. 2017 Nov 30;12(11):e0188896. doi: 10.1371/journal.pone.0188896 (PMC5708728; doi:10.1371/journal.pone.0188896)
Supplement: S1 Table — Results from ICP monitoring in each of the iNPH study patients. (DOCX) [file pone.0188896.s001.docx]

**S1 Table.** **ICP-data from patients with iNPH.**

| **Patients** | **Observations**  **(6-s windows)** | **MWA**  **(mm Hg) (mean)** | **MWA**  **(mm Hg)**  **(SD)** | **CV_MWA_ (%)** | **Mean ICP**  **(mm Hg)**  **(mean)** | **Mean ICP**  **(mm Hg)**  **(SD)** | **CV_MeanICP_**  **(%)** | **HR**  **(/min)**  **(mean)** | **HR**  **(/min)**  **(SD)** | **CV_HR_**  **(%)** | **Pearson correlation**  **(MWA vs HR)** |
| --- | --- | --- | --- | --- | --- | --- | --- | --- | --- | --- | --- |
| 1 | 18.465 | 4.5 | + 1.3 | 29 | 6.7 | + 5.4 | 81 | 68.0 | + 9.9 | 15 | -.15 |
| 2 | 14.796 | 4.3 | + 1.3 | 30 | -1.5 | + 5.7 | 380 | 56.3 | + 8.2 | 15 | .13 |
| 3 | 13.882 | 4.2 | + 1.1 | 31 | 2.3 | + 3.3 | 143 | 66.9 | + 8.8 | 13 | .02 |
| 4 | 21.397 | 3.9 | + 1.0 | 26 | 2.1 | + 6.1 | 290 | 63.7 | + 10.6 | 17 | .02 |
| 5 | 13.881 | 5.0 | + 0.7 | 14 | 1.6 | + 3.3 | 206 | 88.0 | + 9.8 | 11 | .23 |
| 6 | 13.269 | 7.3 | + 1.2 | 16 | 10.4 | + 4.5 | 43 | 61.5 | + 7.2 | 12 | -.09 |
| 7 | 10.530 | 5.5 | + 1.3 | 24 | 2.5 | + 3.4 | 136 | 58.4 | + 6.5 | 11 | -.14 |
| 8 | 12.312 | 2.9 | + 0.8 | 28 | 2.8 | + 2.7 | 96 | 59.9 | + 8.6 | 14 | .14 |
| 9 | 7.997 | 6.1 | + 1.3 | 21 | 3.8 | + 3.3 | 87 | 70.0 | + 7.6 | 11 | .04 |
| 10 | 9.105 | 4.4 | + 0.8 | 18 | 2.7 | + 4.3 | 159 | 78.2 | + 8.3 | 11 | -.10 |
| 11 | 13.562 | 3.4 | + 0.9 | 26 | 2.6 | + 3.1 | 19 | 72.9 | + 12.1 | 17 | -.23 |
| 12 | 12.938 | 2.2 | + 0.9 | 41 | -0.2 | + 8.8 | 4400 | 55.6 | + 11.1 | 20 | .43 |
| 13 | 11.585 | 3.9 | + 1.1 | 28 | 4.5 | + 6.5 | 144 | 62.0 | + 7.0 | 11 | -.08 |
| 14 | 13.464 | 4.7 | + 1.2 | 26 | 6.9 | + 4.7 | 68 | 52.7 | + 10.6 | 20 | -.10 |
| 15 | 13.676 | 4.3 | + 0.8 | 19 | 0.1 | + 5.6 | 5600 | 65.6 | + 7.7 | 12 | -.07 |
| 16 | 13.296 | 6.4 | + 2.2 | 34 | 7.1 | + 3.6 | 51 | 61.8 | + 9.6 | 16 | .09 |
| 17 | 8.465 | 4.4 | + 1.0 | 23 | -4.5 | + 5.4 | 120 | 54.9 | + 8.6 | 16 | .18 |
| 18 | 7.466 | 4.6 | + 1.6 | 35 | 6.9 | + 3.1 | 45 | 57.5 | + 6.9 | 12 | .14 |
| 19 | 15.160 | 5.7 | + 1.3 | 23 | 1.7 | + 3.8 | 224 | 66.8 | + 8.0 | 12 | -.05 |
| 20 | 13.344 | 6.6 | + 1.8 | 27 | 2.8 | + 4.8 | 421 | 76.2 | + 13.7 | 18 | -.46 |
| 21 | 1.501 | 4.1 | + 0.5 | 12 | 3.2 | + 1.9 | 59 | 73.3 | + 6.0 | 08 | -.19 |
| 22 | 9.849 | 3.8 | + 1.1 | 29 | 14.5 | + 8.1 | 56 | 54.6 | + 9.7 | 18 | .29 |
| **All**  **(n = 22) (median with range)** | 13.283  (1501, 21397) | 4.4  (2.2, 7.3) | +1.1  (+.5, +2.2) | 26  (12, 41) | 2.8  (-4.5, 14.5) | +4.4  (+1.9, +11.8) | 128  (19, 5600) | 63.9  (52.7, 88.0) | 8.6  (6.0, 13.7) | 14  (8, 20) | -.02  (-.46, .43) |

**ICP:** intracranial pressure

**iNPH:** idiopathic normal pressure hydrocephalus

**MWA:** mean ICP wave amplitude (pulsatile ICP)

**CV:** coefficient of variance (SD/Mean)

**HR:** heart rate

**SD:** standard deviation
