# Supplementary material for: Non-invasive assessment of pulsatile intracranial pressure with phase-contrast magnetic resonance imaging
Source: PLoS One. 2017 Nov 30;12(11):e0188896. doi: 10.1371/journal.pone.0188896 (PMC5708728; doi:10.1371/journal.pone.0188896)
Supplement: S2 Table — Results from PC-MRI in each of the iNPH study patients. (DOCX) [file pone.0188896.s002.docx]

**S2 Table. PC-MRI data from iNPH patients**

| **Patients** | **MRI-dP**  **(mmHg/cm)** | **ROI area**  **(cm^2^)** | **Number of pixels** | **HR**  **(/min)** |
| --- | --- | --- | --- | --- |
| 1 | .046 | 2.28 | 584 | 69 |
| 2 | .056 | 1.97 | 504 | 52 |
| 3 | .049 | 1.40 | 358 | 76 |
| 4 | .070 | 1.48 | 379 | 54 |
| 5 | .041 | 1.35 | 346 | 71 |
| 6 | .054 | 1.31 | 335 | 70 |
| 7 | .027 | 2.50 | 640 | 56 |
| 8 | .032 | 1.85 | 473 | 62 |
| 9 | .015 | 1.59 | 407 | 66 |
| 10 | .044 | 1.89 | 484 | 76 |
| 11 | .036 | 2.13 | 546 | 92 |
| 12 | .083 | 1.20 | 306 | 56 |
| 13 | .032 | 1.27 | 325 | 61 |
| 14 | .045 | 1.65 | 422 | 55 |
| 15 | .035 | 1.88 | 481 | 71 |
| 16 | .080 | 1.20 | 306 | 73 |
| 17 | .026 | 1.84 | 470 | 56 |
| 18 | .042 | 1.63 | 418 | 69 |
| 19 | .016 | 1.62 | 415 | 64 |
| 20 | .040 | 3.50 | 895 | 81 |
| 21 | .052 | 1.95 | 500 | 75 |
| 22 | .039 | 2.39 | 611 | 58 |
| **All**  **(median with range)** | **.041 (.015, .083)** | **1.74 (1.20, 3.50)** | **446 (306, 895)** | **68 (52, 92)** |

**PC-MRI:** phase-contrast magnetic resonance imaging

**iNPH:** idiopathic normal pressure hydrocephalus

**MRI-dP:** MRI-derived peak to peak pulse pressure gradient

**ROI:** region of interest

**HR:** heart rate
