# Supplementary material for: Non-invasive assessment of pulsatile intracranial pressure with phase-contrast magnetic resonance imaging
Source: PLoS One. 2017 Nov 30;12(11):e0188896. doi: 10.1371/journal.pone.0188896 (PMC5708728; doi:10.1371/journal.pone.0188896)
Supplement: S3 Table — Results from PC-MRI in each healthy subject at each time point. PC-MRI exams (MRI-1 and MRI-2) within one time point (e.g. “Time1”) were performed with the possible closest proximity in time. The distance in time between two time points (e.g. “Time1” and “Time2”) was approximately two hours. (DOCX) [file pone.0188896.s003.docx]

**S3 Table. PC-MRI data retrieved from healthy controls.**

| **Healthy control** | **PC-MRI time point** | **MRI-dP (mmHg/cm)** | **ROI (cm^2^)** | **ROI (pixels)** | **HR** |
| --- | --- | --- | --- | --- | --- |
| 1 | Time1 MRI-1 | .034 | 1.22 | 312 | 63 |
|  | Time1 MRI-2 | .043 | 1.44 | 369 | 65 |
|  | Time2 MRI-1 | .040 | 1.22 | 312 | 75 |
|  | Time2 MRI-2 | .047 | 1.44 | 369 | 73 |
|  | **Median (range)** | **.042 (.034, .047)** | **1.33 (1.22, 1.44)** | **341 (312, 369)** | **69 (63, 75)** |
| 2 | Time1 MRI-1 | .100 | .79 | 201 | 67 |
|  | Time1 MRI-2 | .094 | .72 | 185 | 67 |
|  | Time2 MRI-1 | .092 | .79 | 203 | 64 |
|  | Time2 MRI-2 | .085 | .76 | 194 | 63 |
|  | **Median (range)** | **.093 (.085, .100)** | **.77 (.72, .79)** | **198 (185, 203)** | **66 (63, 67)** |
| 3 | Time1 MRI-1 | .052 | 1.42 | 364 | 61 |
|  | Time1 MRI-2 | .042 | 1.26 | 323 | 52 |
|  | Time2 MRI-1 | .074 | 1.10 | 282 | 54 |
|  | Time3 MRI-1 | .079 | 1.11 | 284 | 57 |
|  | **Median (range)** | **.063 (.042, .079)** | **1.19 (1.10, 1.42)** | **304 (282, 364)** | **56 (52, 61)** |
| 4 | Time1 MRI-1 | .031 | 1.70 | 435 | 50 |
|  | Time1 MRI-2 | .032 | 1.40 | 358 | 51 |
|  | Time2 MRI-1 | .027 | 1.53 | 392 | 45 |
|  | Time2 MRI-2 | .030 | 1.63 | 417 | 48 |
|  | **Median (range)** | **.031 (.027, .032)** | **1.58 (1.40, 1.70)** | **405 (358, 435)** | **49 (45, 51)** |
| All | **ICC (95 % CI)** | **0.884 (0.587, 0.991)** | **0.884 (0.597, 0.991)** | **0.883 (0.593, 0.991)** | **n/a** |
|  | **Median (range) (n=4)** | **.053 (.031, .093)** | **1.26 (.77, 1.58)** | **323 (198, 405)** | **61 (49, 69)** |

**PC-MRI:** phase-contrast magnetic resonance imaging

**MRI-dP:** MRI-derived peak to peak pulse pressure gradient

**ICC:** Intra-class correlation coefficient

**ROI:** region of interest

**HR:** Heart rate
